# Engaging U.S. Adults with Serious Mental Illness in Participatory Design Research Exercises

**DOI:** 10.3390/ijerph19116743

**Published:** 2022-05-31

**Authors:** Kimberly A. Rollings

**Affiliations:** 1 School of Architecture, University of Notre Dame, Notre Dame, IN 46556, USA; kirollin@umich.edu; 2 Department of Psychology, University of Notre Dame, Notre Dame, IN 46556, USA; 3 Health and Design Research Fellow, Institute for Healthcare Policy and Innovation, University of Michigan, Ann Arbor, MI 48109, USA

**Keywords:** Clubhouse Model, adults, mental health, mental illness, serious mental illness, built environment, architecture, participatory design research

## Abstract

Clubhouses are non-clinical, community-based centers for adult members with serious mental illness. The evidence-based model assists adults with identifying employment, housing, education, and social opportunities; wellness and health-promoting activities; reducing hospitalizations and criminal justice system involvement; and improving social relationships, satisfaction, and quality of life. The model enables member participation in all Clubhouse operations, yet offers little guidance concerning facility design and member engagement in the design process. This case study explored the use of participatory design research exercises to (1) document member needs, preferences, and priorities to inform the design of a new midwestern U.S. Clubhouse facility and (2) meaningfully engage members (*n* = 16) in the design process. Four participatory design research exercises were developed, administered, and analyzed. Results revealed aesthetics and ambience; safety and security; ease of use and maintenance; adaptability, flexibility, and accessibility; and transportation as future priorities. Space and furnishing needs and priorities were also identified. Informal observations and participant feedback suggested that the participatory exercises meaningfully engaged members in a manner aligned with Clubhouse Model principles by centering member dignity, strengths, and work-oriented expectations. Future directions for research on Clubhouse design and member engagement in the design process are also discussed.

## 1. Introduction

Clubhouses are non-clinical, community-based centers for adult members diagnosed with serious mental illness [1]. The evidence-based Clubhouse Model assists members with employment, housing, education, social, and health-promoting activities, and requires staff work with members on all Clubhouse matters as equals [1,2]. Despite this inclusive requirement and an increasing recognition of the importance of participatory processes [3,4], the Clubhouse Model offers no guidance concerning engaging members in the Clubhouse design process [2]. The model also offers little guidance on facility design and location, overlooking existing literature on beneficial mental and behavioral health facility design features important for recovery. The purpose of this case study was to explore the use of participatory design research exercises to (1) document member needs, preferences, and priorities relating to the design of a new midwestern U.S. Clubhouse facility and (2) meaningfully engage members in the design process in alignment with Clubhouse Model principles. The following background sections provide context about mental illness in the U.S., the Clubhouse Model, mental and behavioral health facility design, and Clubhouse facilities.

## 2. Background

### 2.1. Mental Illness and Serious Mental Illness in the U.S.

Mental illness affected one in five (21%; 52.9 million people) U.S. adults according to 2020 data [5,6]. Serious mental illnesses (SMIs), including major depression, schizophrenia, bipolar disorder, obsessive compulsive disorder, panic disorder, post-traumatic stress disorder, and borderline personality disorder, affected one in 20 adults (5.6%; 14.2 million people) [5,7]. Only an estimated 46% and 65% of adults with mental illness and SMI, respectively, received treatment that year [5]. People often experience long delays before seeking or obtaining help due to delayed diagnoses, no insurance coverage (11% of adults), and a lack of available mental health professionals (55% of counties lack a psychiatrist; 142 million residents reside in mental health professional shortage areas) [5,8,9,10,11]). Treatment delays contributed to associations between mental illness and incarceration (2 in 5), unemployment (90% in some states), homelessness (25–30%), and substance abuse (35%) [5,12,13,14,15,16,17,18]. Moreover, 46% of people who died by suicide had a diagnosed mental health condition [19], while an estimated 90% displayed symptoms of a mental health condition [20]. Other adults with SMI living in society are frequently isolated and confined to their homes, with many living at or near the federal poverty level. All face the stigma of mental illness and a lack of appropriate support across the phases of recovery. The Clubhouse Model contributes to addressing these gaps for adults with SMI already living in society.

### 2.2. The Clubhouse Model of Psychosocial Rehabilitation

The Clubhouse Model of Psychosocial Rehabilitation originated at the Fountain House in New York City in the 1940s and 1950s [1,21]. The model recognizes that recovery from SMI requires addressing the whole person, and a supportive community. Clubhouses are formally established, non-clinical, work-ordered communities of adults diagnosed with SMI, and staff. Unlike medical treatment models that focus only on SMI and disability, Clubhouses are strengths-based and focus on what members—not patients—can accomplish, helping them lead socially satisfying, vocationally productive lives via restorative work and work-mediated relationships [1,22]. Community, independence, and hope [23] in an environment that promotes work and creates opportunities for socially supportive connections [24] are critical to recovery and the success of the Clubhouse Model [25]. Clubhouse International (CI) coordinates the development and support of all Clubhouses and training, including internationally. In the U.S., the Clubhouse Model is recognized as an evidence-based practice by the U.S. Substance Abuse and Mental Health Services Administration (SAMHSA). Clubhouse effectiveness and quality improvement research is conducted by The Program for Clubhouse Research at the University of Massachusetts Medical School [1].

Key components of the Clubhouse Model are the right to membership, meaningful relationships, and lifetime re-entry; the “need to be needed”; a choice in Clubhouse staff and work activities; and access to all Clubhouse services [1,22,26]. Available services and activities include assistance identifying employment; safe and affordable housing and medical services; supported education; social and recreational opportunities; health promotion and wellness activities; outreach and advocacy; community support services; and crisis intervention [1,27]. The inclusive model requires that participants, referred to as members [28], be permitted to contribute to all Clubhouse operations, decision-making, and governance by working side-by-side with Clubhouse staff as equals following a structured, rehabilitative work-ordered day [29,30,31]. Voluntary and free Clubhouse memberships are available to adults with SMI who are at least 18 years of age and offer equal access to all Clubhouse resources [32,33].

Considering that mental illness costs the U.S. at least $300 billion annually in healthcare expenditures, disability benefits, and lost productivity [34,35], Clubhouses contribute to addressing gaps in the continuum of mental health care. A systematic review of randomized control trials indicated that the Clubhouse Model led to improved employment rate, tenure, and wages; better quality of life, satisfaction, and self-esteem; and reductions in the number and duration of psychiatric hospitalizations and emergency mental health encounters among members [1]. Quasi-experimental and observational study results showed improvements in social relationships, social inclusion, education, and increased health promotion activities, but more research is needed in these areas [1]. Other work found that criminal justice system involvement was substantially diminished during and after Clubhouse membership [36]. Literature that examined cost effectiveness further demonstrated that the Clubhouse Model reduced state and local government costs from hospitalization, incarceration, and institutionalization [37,38]. The following sections describe additional existing literature on design and the built environment relevant to mental and behavioral health facilities and Clubhouse facilities.

### 2.3. Mental and Behavioral Health and the Built Environment

#### 2.3.1. Mental and Behavioral Health Facility Design

The physical quality of any treatment or rehabilitation environment plays a significant role in the recovery process of adults with SMI, and mental and behavioral issues more broadly [39,40,41,42,43,44,45]. Literature examining effects of the built environment on mental health documented associations between features, such as lighting, views, daylight, layout, crowding, and noise, and outcomes relevant to members, such as safety, cognitive functioning, productivity, stress, depression, behavioral disturbances, and psychosocial processes (e.g., control, social interaction, privacy, and restoration from stress and fatigue) related to mental health [46,47,48,49,50,51,52]. Other literature on mental and behavioral health (MBH) facilities found that well-maintained, residential, and homelike—rather than institutional—environments, safety, security, aesthetics, attractive furnishings, daylight, and visual and physical access to nature were associated with positive outcomes including addressing psychological needs, satisfaction, and perceptions of care [42,43,44,52,53,54,55,56,57] among patients, residents, and staff. Environments designed to facilitate control, choice, accessibility, social interaction, and a sense of community were also associated with positive benefits [42,52,53,54]. Much of the MBH literature focused on inpatient, outpatient, and residential settings and not Clubhouse environments. Clubhouses are work-ordered community centers and not clinical or residential facilities. The effects of Clubhouse environments on adults with SMI engaged in a work-ordered day are less understood. Additionally, most of the MBH literature was conducted with providers, staff, and design professionals and not adults with mental and behavioral issues.

#### 2.3.2. Clubhouse Facility Design

Today, nearly 300 Clubhouse facilities in 33 U.S. states and 30 countries serve more than 55,000 people [1]. Entities working to open a Clubhouse are supported by Clubhouse International (CI) that oversees the International Standards for Clubhouse Programs and a certification process. Standards contain eight categories: Membership, Relationships, Space, Work-Ordered Day, Employment, Education, Functions of the House, and Funding, Governance, and Administration [2]. These standards, however, only offer the following three design guidance items for Clubhouse facilities:

“Space emphasizes the importance of creating a dignified, attractive environment where important work is carried out.

- 12. The Clubhouse has its own identity, including its own name, mailing address, email, and telephone number.
- 13. The Clubhouse is located in its own physical space. It is separate from any mental health center or institutional settings, and is impermeable to other programs. The Clubhouse is designed to facilitate the work-ordered day and at the same time be attractive, adequate in size, and convey a sense of respect and dignity.
- 14. All Clubhouse space is member and staff accessible. There are no staff only or member only spaces” [2].

While CI standards do describe deinstitutionalized, dignified, and attractive environments that are adequate in size, they offer little guidance on design and site selection relative to other features associated with positive outcomes. Standards also exclude guidance on how to include members (and staff) in the planning and design of a Clubhouse facility. The strong social relationships facilitated by the Clubhouse environment empower members and help them pursue recovery goals. These relationships are developed through the work-ordered day and employment opportunities likely influenced by Clubhouse design and location. The work-ordered day engages members in operations such as reception; clerical work; food preparation; financial, retail, and transportation activities; cleaning and maintenance; identifying medical, psychological, pharmacological, and substance abuse services; and daily “reach-out” to stay in contact with absent members [2]. Clubhouses also facilitate social interaction by hosting daily, evening, weekend, and holiday programming and events. These activities, along with the needs of adults with SMI, present unique design challenges that must be identified and addressed in the design process.

The physical location of a Clubhouse facility can also influence Clubhouse operations and connections in the surrounding community, especially relating to employment. Clubhouses help members seeking employment with their job search and career development by identifying transitional, supported, and independent employment as described elsewhere [1]. Staff develop and maintain relationships with employers to secure positions for members, provide member job training and support, and send a Clubhouse staff or alternate member in the event of an absence [1,58,59]. This “absence coverage” incentivizes nearby employers to participate in Clubhouse employment programs. Clubhouse facility location influences the quantity and types of nearby employment opportunities, staff ability to develop trusting relationships with employers, and employee ability to walk or take public transportation to jobs, housing, educational opportunities, groceries, and other resources. Considering the importance of MBH environments to recovery; that CI standards require member participation in all Clubhouse operations; and that members are the main Clubhouse user group, this study aimed to document member perspectives on Clubhouse design and to engage members in the design process. A participatory approach was used as members accustomed to the work-ordered day are uniquely positioned to inform the Clubhouse design process. The present study explored use of participatory design research exercises aligned with Clubhouse Model principles to answer the following research questions:

- 1. What are Clubhouse members’ needs, preferences, and priorities relating to the design of a future Clubhouse facility?
- 2. Are participatory design research exercises a potentially meaningful and effective mechanism by which to engage Clubhouse members in the design process?

## 3. Methods

### 3.1. Research Design

This case study was participatory in two ways: by engaging members as partners in exploring their knowledge of and experiences with Clubhouses and mental illness; and by designing the data collection process to engage members and the research team collaboratively as equals, rather than studying members only as the “subjects” of research [4,60]. Participatory design actively engages all project stakeholders and end users in the design process to improve design solutions and outcomes such as user satisfaction and quality of life. Participatory approaches are often used, including in design practice and mental health research, to better understand user needs and experiences, address the needs of under-served populations, or prompt systems-level change [4]. Who participates, how and when participants are engaged in the research process, and the purposes that are served by participation determine whether the research is participatory [3,4]. Three phases typically occur in a participatory design process: identifying user needs, iterative design, and evaluation [3]. This study focused on phase one and identifying user needs to meaningfully engage Clubhouse members early in the design process in a manner consistent with Clubhouse Model principles.

The term, “participatory design research exercises”, is used in this paper as the research team’s role was only to focus on phase one of the participatory design process and gather data to answer project stakeholder questions prior to beginning the design process. Four participatory design research exercises documented member needs, preferences, and priorities. Research team observations of and feedback from participants and project stakeholders were also documented to reflect on the usefulness of the information collected, member engagement in the design process, and alignment with the Clubhouse Model. The following sections describe the project context and development, administering, and analysis of the participatory design exercises. Exercises were conducted as part of a larger university-community partnership also briefly described in the next section.

### 3.2. Project Location and Context

A local “Clubhouse Working Group” (hereafter, “Group”) formed in a midwestern U.S. city of approximately 100,000 people. The Group coalesced to open a new Clubhouse after the local non-accredited facility and its affiliated mental health provider closed. At least 20 people, including former Clubhouse members, staff, and family members; mental health providers; local National Alliance on Mental Illness chapter members; and other interested community members joined the Group. Meetings were held bimonthly, and subgroups formed to focus on fundraising, budgeting, mission statements, and preparing for CI certification and meetings. The Group collaborated with two local university faculty and established a university-community partnership that informed mission statement development, fundraising efforts, research, site identification, and new facility design. One architecture studio faculty instructor facilitated an undergraduate design project that generated proposals for a new local Clubhouse. Proposals highlighted location and building design issues for consideration by the Group, and were later used in participatory design research exercises facilitated by the author.

The author also formally joined the Group and became a research collaborator. This participation consisted of attending bi-monthly meetings, informing mission statement development and fundraising efforts, lecturing to and serving as a design critic for the students’ studio project; proposing and coordinating participatory design research exercises; informing Clubhouse site selection and building renovations; and securing a grant to support collaborative research related to Group work. Research activities included overseeing a community mental health needs assessment; informally reviewing literature relevant to Clubhouse design, the built environment, and mental health; observing two existing Clubhouse facilities; documenting U.S. Clubhouse facilities (photos and available services) via systematic online research; and conducting research on local Clubhouse transportation needs.

### 3.3. Participants

Seventeen former Clubhouse members voluntarily attended the participatory session (16 submitted written questionnaires; 17 participated in design project feedback and space prioritization exercises). The Group emailed and called an existing, well-connected network of former members to share information about the project and encourage attendance at the session among those interested in participating. The session was scheduled to occur during an optional regular monthly meeting of former members. Transportation was coordinated by the Group, and dinner was arranged by the Group but funded by the research team. Three Group members, the author, and three undergraduate research assistants facilitated the participatory session. The study was approved by the Group and the University of Notre Dame Institutional Review Board.

### 3.4. Procedures

#### 3.4.1. Participatory Exercise Development

Participatory design exercises were used to document Clubhouse member needs, preferences, and priorities for two reasons. First, former Clubhouse members felt “in the dark” concerning the Group’s progress towards opening a new Clubhouse. The Group, which included two former members, wanted to engage more members in the process. Methods used, however, needed to align with Clubhouse Model principles by centering member strengths, dignity, and work-oriented expectations. Voluntary participation also still needed to afford choice, control, and ownership. Second, to engage as many attendees as possible, methods had to accommodate a range of reading, writing, and speaking abilities; comfort levels with verbally communicating with others, and willingness to share information. A participatory approach addressed both reasons.

An informal literature review, Clubhouse research and site visits, and Group discussions informed the development and testing of four data collection exercises: a questionnaire, review of student Clubhouse design proposals (building floor plans, elevations, and perspective renderings), a space prioritization exercise, and an ideal Clubhouse drawing. Additional exercises were developed and tested, but not selected for use due to redundancy of data collected or potential lack of member engagement and task completion. The questionnaire (see Form S1) was developed with the Group based on their questions about former Clubhouse facility spaces and activities, meetings with Clubhouse International, and interactions with members. The questionnaire collected information from individuals about former Clubhouse experiences before they were influenced by peers and subsequent exercises. The Group expressed concern that responses might focus too much on the former rather than future Clubhouse design. Twenty-two primarily open-ended items remained after piloting by the research team. Piloting among Group members and students examined whether items were appropriate for a variety of reading levels and that responses contained the desired information. Items inquired about former Clubhouse experiences including time spent there and in what spaces; transportation method and needs; services offered and used; shared spaces; favorite and least favorite spaces and activities; needed services; responsibilities and what helped or hindered completing them; and what differentiated the Clubhouse from a healthcare facility. Four additional and optional items requested age, gender, education level, and mental illness diagnoses to enable assessment of the representativeness of participating Members.

The second exercise solicited member feedback on completed student Clubhouse design proposals. Six fourth-year undergraduate architecture students’ hand-drawn and water-colored projects were selected for presentation based on a variety of proposed site and building attributes, image clarity, and completion. The studio professor and students agreed to allow the research team to present their work as part of the participatory session. This exercise introduced members to the design process and how to read architectural renderings; presented design and location ideas for member and Group consideration; and allowed members to share questions and ideas. Project images also served as prompts for discussion about space and activity needs, preferences, and priorities. Feedback was written on “sticky notes” and physically attached to the drawings, offering a tangible task for members to complete. This exercise approach is often used by practicing design professionals when gathering community input and feedback.

The third exercise required small groups of members to prioritize the types of spaces needed in the new facility. A list of possible spaces was created based on other Clubhouse facilities, Clubhouse literature, and discussion with the Group. Identifying and prioritizing the number and types of spaces needed is a common architectural programming exercise completed with clients. Programming includes the collection and analysis of information about building, facility, or setting requirements to help clients make decisions and inform the design process [61]. Members were asked to sort a stack of cards, each containing the name of one type of space (e.g., meeting room), into “must-,” “should-,” and “could-have” stacks (see Form S2) according to the following definitions:

- Must: A required space, without which a new Clubhouse could not function.
- Should: Needed in a new Clubhouse, but could be eliminated if necessary.
- Could: Would be very nice to have, but is more of a luxury.

The fourth exercise encouraged members to draw their ideal Clubhouse facility. This creative activity required no speaking or interaction to complete if desired, but also invited members to optionally present their completed work to each other. The four exercises facilitated varied levels of engagement and interaction with peers, staff, and the research team, and collected various amounts and types of data. Exercises were intentionally ordered to “build” the amount of engagement and data collected, with each exercise providing information or “prompts” for the subsequent activity. With the exception of the questionnaire, work-oriented exercises assigned tangible tasks to attendees to complete within a fixed amount of time. The variety of exercises offered also allowed the research team to collect the same data in multiple ways to improve study reliability and validity.

#### 3.4.2. Data Collection

**Figure 1:**
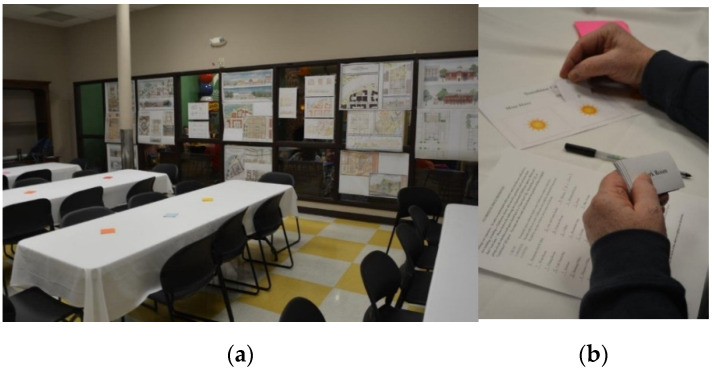
Participatory design research exercise session set-up. (**a**) Six Clubhouse design proposals created by students were posted on the glass wall in front of four long folding tables. (**b**) A Clubhouse member completes the space prioritization exercise by sorting spaces according to whether they “must,” “should,” or “could” be included in a new Clubhouse.

Prior to hosting the 90-min participatory session, the research team read and attended lectures about Clubhouses and mental illness; received training on data collection and assisting study participants without influencing their responses; and were introduced to and practiced using inclusive language. Students were also trained to note observations during the session concerning participant responses to or comments about the exercises, challenges encountered, and ideas for improvement of the exercises. The session was held in a private room at a local downtown café near where former facility staff and members met monthly since the local Clubhouse facility closed. The space was arranged with 8 chairs surrounding each of four folding tables covered by tablecloths. Student design proposals were posted along one wall and colorful “sticky notes” were placed on each table (Figure 1a).

Attendees were greeted by familiar Group personnel and introduced to the research team, all of whom wore nametags. Members were encouraged to eat dinner while completing a questionnaire and waiting for others to arrive. The research team distributed questionnaires and writing utensils, briefly explained instructions, emphasized that participation was voluntary, and stated that sharing information throughout the evening was optional and that the Group and research team were available for questions. Because no identifying information was collected from participants in the public café, formal written consent was not required of participants. Verbal consent was obtained from members by the Clubhouse Group during recruitment. The Group also confirmed that all attendees were informed by phone call or in-person about the session.

The session formally began with research team introductions and explanation of the evening’s purpose and agenda. Emphasis was placed on member expertise and the value of their input to inform the design and success of a new Clubhouse facility. An undergraduate architecture student who participated in both the studio and research group then introduced the studio project and second exercise. The author explained and demonstrated how to view and discuss the images on the wall and provide feedback using sticky notes. Members were then invited to view the projects, write comments on sticky notes, and attach their feedback to the images for 20 min. Members who had difficultly standing, walking, or talking were aided by staff and the research team so all could participate. Members were then divided into groups of three or four people and asked to complete the space prioritization exercise in another 20-min period (Figure 1b). Finally, members were invited to draw their “ideal” Clubhouse building using colored-pencils and markers, and present their work to their table in the remaining time.

#### 3.4.3. Analysis

The research team met after the session to inventory collected exercise materials and debrief. Research team observations noted during the session were aggregated, discussed, and later used during coding and interpretation when discrepancies arose. Written questionnaire responses, sticky note text, and space prioritization exercise results were manually copied into three spreadsheets by pairs of research assistants to maintain accuracy. Questionnaire responses were counted and space priority ratings were averaged. Sticky note text was coded to identify and organize responses by space and topic. Due to the small number of completed ideal Clubhouse drawings, images were excluded from analysis (see 4. Results). The remaining three exercises were then analyzed in summation to identify overall topics relating to member needs, preferences, and priorities for future Clubhouse design. This analysis was iteratively completed by the author and three research assistants until consensus was reached. The Group declined to participate in analysis and interpretation of results. A draft results summary was sent to the Group, including two participants and two staff attendees of the participatory exercises, for feedback. They approved the results report “as is” and offered no feedback.

## 4. Results

### 4.1. Participant and Former Clubhouse Characteristics

#### 4.1.1. Participant Characteristics

Sixteen attendees between 39 and 72 years of age (5 males, 9 females, 2 not reported) submitted questionnaires at varying levels of completion. Twelve former and three future Clubhouse members completed high school (4), some college (9), or a college degree (2; 1 not reported). Mental health diagnoses, as written by members, included: attention deficit hyperactivity disorder, anxiety, bipolar disorder, borderline personality disorder, depression, major depression with psychosis, manic depressive, personality disorder, post-traumatic stress disorder, chronic undifferentiated schizophrenia, and schizoaffective disorder. Seven members reported multiple diagnoses, three reported having one, and six opted not to share diagnoses.

#### 4.1.2. Former Clubhouse Use and Characteristics

Attendees reported spending time at the former Clubhouse 4–6 days per week, with many often spending all day there. According to members, available Clubhouse services (Table S1a) included meal preparation and serving, clerical and office duties, education activities, accounting, maintenance, housekeeping, recreational and social events, a thrift store and snack bar, employment and housing assistance, outreach, artistic activities, case management, gardening, health and counseling services, transportation, and a library. Meal preparation and serving, clerical and accounting work, educational activities, maintenance, and cleaning were most common. Member responsibilities (Table S1a) involved working with computers (menus, attendance records, newsletter, statistics, classes), retail (cashier and inventory) and education tasks, meal preparation and serving, fundraising, banking, teaching, and leadership. Table S1b summarizes responses concerning what made fulfilling Clubhouse member responsibilities easier or more difficult. Having adequate help, space, and discussion made completing tasks easier. Simplifying maintenance tasks was also important and a recurring topic across exercises. A lack of space, supplies, technical support, and help impeded work.

### 4.2. Research Question 1: Member Needs, Preferences, and Priorities

**Figure 2:**
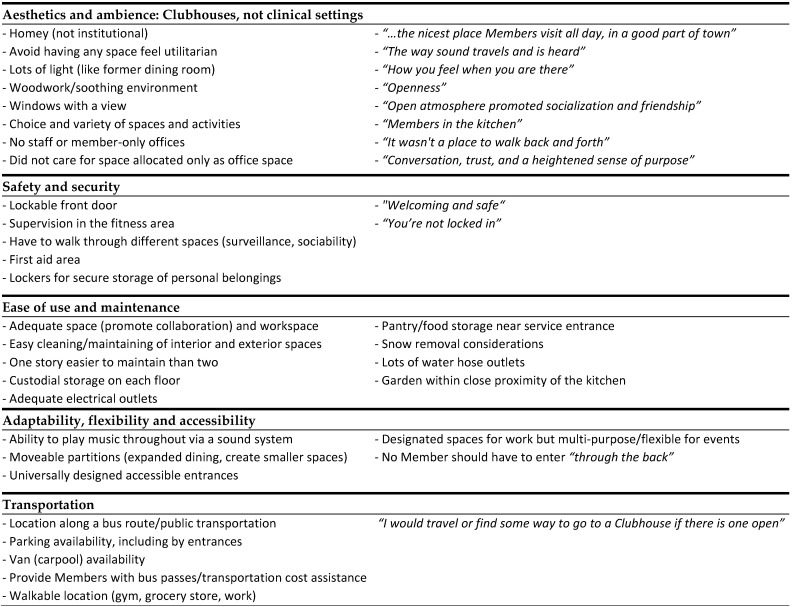
A summary of Member responses to participatory exercises.

Figure 2 identifies the topics that emerged from the research team’s synthesis of questionnaire responses, design project feedback, and the space prioritization exercise. The following sections describe each topic in detail. With the exception of the ideal Clubhouse drawings, descriptive results from individual participatory exercises can be found in the Supplementary Materials (Tables S1a–d and S2). By request of the Group, all responses are represented in each table to place equal value on individual Members’ responses and experiences shared. Although several members drew and labeled an ideal Clubhouse building or floor plan, only six attendees submitted final drawings. Review of the drawings revealed no new information or discrepancies compared to other exercise results so drawing analyses results are not reported.

#### 4.2.1. Aesthetics and Ambience: Clubhouses, Not Clinical Settings

Many member comments related to Clubhouse aesthetics and ambience (Figure 2). One quote often shared by members during the session, as well as the Group during bi-monthly meetings, was that a “*Clubhouse should be the nicest place Members visit all day, in a good part of town*”. Participant responses and discussions noted by the research team also highlighted the distinction between a Clubhouse and a clinical healthcare setting. Window views, light, openness, and member accessibility to all spaces were emphasized. Desired architectural features included incorporating local styles into the design of the Clubhouse building and maintaining some of the details from the old building, such as the “*soothing woodwork*”. Other responses relevant to this topic were generated by questionnaire items that asked members to describe what makes completing their Clubhouse responsibilities easier, and what makes a Clubhouse facility different from a healthcare facility:

- *“The way sound travels and is heard”*
- *“How you feel when you are there”*
- *“It wasn’t a place to walk back and forth”*
- *“Conversation, trust, and a heightened sense of purpose”*
- *“Knowing that I was making a difference”*
- *“Helping others: It kept me out of my own head.”*
- *“I took on a role of trust both with the staff and the members”*

Additionally, having a choice of spaces, a variety of activities and responsibilities, being able to “*help out*” where needed, helping other members, feeling needed, and being trusted were all important features to members. Environmental qualities that were not desired, based on experiences in prior Clubhouse spaces: the “*dark, stinky, and lonely*” storage garage, “*dull*” commercial kitchen, bathrooms requiring cleaning, and, for “*anti-smokers*”, the smoking area (Table S1d). Member responses articulated, in their words, the importance of the environment to recovery.

#### 4.2.2. Safety and Security 

Members also emphasized safety and security (Figure 2). They stated that Clubhouse environments are welcoming and safe, with a lockable front door, lockable storage for personal belongings, and a first aid area. The layout is open and connected so it requires passing through multiple spaces when circulating to promote natural surveillance and communicating with one another.

#### 4.2.3. Ease of Use and Maintenance 

Ease of use and maintenance were mentioned often in member responses (Figure 2). Having adequate space for work-ordered day responsibilities, food storage located near the service entrance to store commercial kitchen supplies, adequate and appropriately located custodial supply storage, and ample electrical outlets were member priorities, likely due to the former Clubhouse lacking in some of these areas. Members also expressed concern about design features that could complicate maintenance tasks. Work-ordered day responsibilities for members included cleaning, landscaping, basic maintenance, and upkeep. Members responsible for outdoor Clubhouse maintenance pointed out that having a two-story building required more maintenance overall, more equipment, more storage for that equipment, and more risk for those doing exterior maintenance work due to having to climb a ladder. Because a one-story building would require more land to accommodate the same Clubhouse square footage requirements of a two-story building, this consideration was highlighted for the Group and future architect to address.

#### 4.2.4. Adaptability, Flexibility, and Accessibility 

Member comments also related to Clubhouse adaptability, flexibility, and accessibility (Figure 2). Responses described the need to designate spaces for work-ordered day activities (e.g., meeting room, dining area), but to also have adequate amounts of space and multipurpose spaces for “working groups” and activities. Moveable partitions that enable flexibility space sizes were noted. Responses also indicated that some Clubhouse activities shared spaces: the thrift store and snack bar; dining room and multipurpose space; and computer and meeting rooms. There generally were not issues with sharing spaces when “*scheduling was on time and enough computers were available*”. Identifying spaces appropriate for multipurpose use was helpful for the Group to inform the architect and address budget constraints. Accessible entrances and accessibility overall were also priorities (no member should have to enter “*through the back*” for any reason). Additionally, the ability to play music in multiple spaces from one central location was also desired.

#### 4.2.5. Transportation

Transportation was also of concern to Members (Figure 2). Members reported traveling to the former Clubhouse most often by vehicle, via carpool or shuttle service, and public transportation (bus). Two members rode a bicycle at times, but no one walked. They also shared suggestions for improving transportation to the future Clubhouse. While one member said “*I would travel or find some way to go to a Clubhouse if there is one open*”, others felt that locating the Clubhouse along a bus route, providing members with bus passes or assistance with transportation costs, and having a Clubhouse van or car pool would ease transportation difficulties. Local public transportation was limited, especially “after hours” and on Sundays when there was no bus service. Other responses indicated that weather frequently made transportation difficult. Design project feedback also expressed preferences for nearby walkable destinations such as workplaces and a grocery store.

#### 4.2.6. Future Clubhouse Spaces and Furnishings 

**Figure 3:**
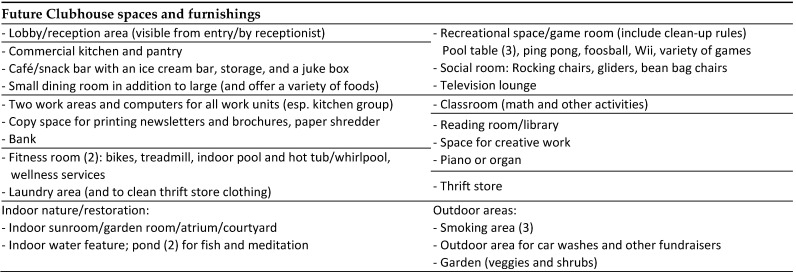
Future Clubhouse space and furnishing preferences. Numbers indicate the quantity of Member responses including that item. No number indicates that there was one Member response.

Member responses regarding needs, preferences, and priorities for different types of spaces and furnishings are summarized in Figure 3. The initial questionnaire asked members about their favorite and least favorite activities and spaces from the former Clubhouse facility, as well as the reasons for their responses (Table S1c,d). One member appreciated the entire building and said, “*I would do anything to get out every day*”, meaning that they would prefer to work on any Clubhouse activity in any space to avoid being isolated at home. The entrance lobby was a favorite space mentioned in all exercises. Members described the importance of entering a Clubhouse and immediately being greeted by a person to feel welcome, as well as giving tours to show visitors “*how we feel about Clubhouse*”. Some members preferred active spaces such as the dining room where “*there was a lot of conversation, eating, and caring*”, while other favored smaller spaces, such as the kitchen, with fewer people and being less “*overwhelmed*”. This information further emphasized that members prefer to have their choice of a variety of sizes and types of spaces. Members’ least favorite activities included cleaning, housekeeping, maintenance activities, cookouts, “*talking in front of large groups*”, and “*not having much to do*”. These responses supported the need for ease of use and maintenance and providing a variety of spaces and activities to enable members to choose and control their level of engagement in activities and with other.

Members also shared preferences for improving upon former and adding new spaces to the future Clubhouse: adding a coffee or sandwich shop with an ice cream bar and juke box, additional dining and work areas, banking services, a well-equipped fitness area and wellness services, laundry for personal and thrift store clothing, and a variety of recreational spaces for communal (games, television, music) and quiet (reading, creative work) activities. Connection to nature and the outdoors was also desired. Responses ranged from ensuring each proposal had the basics to imagining indoor swimming pools, ponds for meditation and fish, gardens, and indoor and outdoor green spaces. Suggested outdoor spaces included an area for car washes and fundraisers, a garden, and a smoking area. After realizing that student projects eliminated smoking areas from proposals due to health concerns, one member emphatically shared that smoking areas were essential and placed a sticky note reading, “*where’s the smoking area*?” on every page of each project. The member and a staff person explained that many members were also recovering from a substance use disorder, or lost everything due to mental illness. Smoking served as one of few “outlets” for some members and required a specific area for that activity to respect members and staff who did not smoke.

#### 4.2.7. Clubhouse Space Priorities 

Table 1 presents the average space priority ratings assigned by members to each type of space. This exercise occurred after the questionnaire and design project review. The first two exercises asked members to describe space and activity preferences based on former Clubhouse experiences and their ideals for a future Clubhouse. The space priorities exercise, on the other hand, required members to prioritize or “narrow the list” in anticipation of budgetary constraints. With few exceptions, the spaces identified as “must have” spaces necessary for a Clubhouse to open were consistent with essential spaces of the prior building and Clubhouse Model standards (e.g., meeting room, lobby/reception, kitchen, copy area, restrooms). Basic Clubhouse operations could occur in these “must have” spaces. From an architectural perspective, storage, mechanical space, and a delivery area were the only required spaces not considered “must have” spaces. Of note was that no outdoor spaces reached “must have” status, even when the average priority rating of all five outdoor space types was calculated (2.1 for covered outdoor space, porch, garden, any outdoor space, and balcony). Having a covered outdoor space was most preferred, followed by a porch and garden. Although members were encouraged to add their own space types to the list provided, none were submitted to the research group.

### 4.3. Research Question 2: Participatory Design Research Exercise Potential

The effectiveness and meaningfulness of the participatory exercises were documented by written research team observations and informal member and Group feedback. This section describes feedback from members, the group, and architecture students (Table 2), feedback focused on each of the participatory exercises, and general research team reflections on preparing and administering the exercises. Members shared that the participatory approach was more appealing and engaging than paper forms and just “*talking to people*” (interviews and focus groups). The variety of exercises and optional levels of engagement also allowed for individual control of participation level. Some Members were fully engaged from the start while others increased participation as the evening progressed (e.g., the person who did not submit a questionnaire). One member stated via e-mail that:

“*Everyone was so engaged in the activities that it truly was a Clubhouse Model event. I don’t think there was a single person who didn’t feel like they weren’t contributing to help with the design of the new Clubhouse*…”

The work-oriented tasks created a sense of need, ownership, and value in alignment with Clubhouse Model principles. Additional feedback from members is included in subsequent sections focused on each of the participatory exercises.

The Group was pleased with the amount and quality of information gathered, and later shared that it informed discussions with members and design professionals. They were also “*thrilled*” with the level of member engagement, the dignity with which members were treated, and the inclusion of members in at least part of the design process. The results summary also provided a physical document that could be referred to by members and the Group in future meetings, especially when conflicts about space priorities arose.

Architecture students who created the design proposals were surprised by the amount of information available from the literature and members to inform supportive design. The students (and faculty) had never designed a Clubhouse or a building for users with mental illness. One student said they would “*completely change [their] design*” after talking to members and researching Clubhouses rather than only architectural precedents (they were not aware of the MBH literature). Another was surprised that the architectural profession does not regularly engage all building occupants in the design process and discussed the importance of including under-represented user groups. Student feedback suggested that future use of the approach in professional practice overall, and with Clubhouse members and stakeholders, was worthwhile. Students also reported being motivated to design well once they interacted with Clubhouse members, stating that, “*We did so little but it mattered so much*”.

#### 4.3.1. Questionnaire

Although the questionnaire documented useful descriptive data, greeting members with a “*form to fill out*” was not the most welcoming or engaging way to begin the session, based on observations and member and Group feedback. The questionnaire was lengthy and required “*a lot of reading and writing*”. Feedback and several missing responses to items suggested that the length, and potentially the reading and writing skills required, inhibited some members’ responses. A more engaging format, or perhaps a brief, “ice-breaker” activity may have been more appealing. The questionnaire, however, was members’ only opportunity to share their individual responses without peer influence and allowed participants who did respond to express themselves in ways that were not captured by other exercises.

#### 4.3.2. Design Project Feedback

Members commented that they were impressed by the students’ work and inspired by the ideas presented during the session. One member shared via email after the session that “*…this type of visualization would have never been possible without [student] skill and imagination. Thank you ever so much for this gift*”. According to the Group, reviewing Clubhouse design proposals enabled identification of a more robust list of member design needs, preferences, and priorities than prior Group discussions with members. Instead of focusing only on the previous Clubhouse environment, student project images prompted discussion of more design attributes. The research team suggested that future exercises might—instead of or in addition to student projects—present images from existing Clubhouses and ensure representation of design elements identified in the MBH literature to generate more comprehensive data for design professionals.

#### 4.3.3. Space Prioritization

The space prioritization exercise was the most engaging and enjoyable according to observations and member and Group feedback. One member wrote that, “s*omehow, the physical act of placing that slip of paper into a slot carried just a little more weight*”. The head of the Group, with teary eyes, shared that she had never seen members so engaged or approach a task with such dedication. Another member shared that the task felt “*work-ordered*” and important. Lively discussions about space priorities occurred. Several members asked for more time and to add spaces to the list. Members were overheard discussing why luxury items like a swimming pool constantly requested by some Members were not “must have” items because an elevator or lift was needed for members who use a wheelchair or other mobility devices. A fitness room was clearly not important to some members, while a smoking area continued to be a priority for smokers. Members decided, amongst themselves, what spaces to exclude instead of have them “taken away” later in the process by others. The process afforded control and ownership to members that felt otherwise excluded from new Clubhouse discussions. Other members said they would refer to this exercise whenever conflict occurred about new Clubhouse space priorities.

#### 4.3.4. Ideal Clubhouse Drawings

The ideal Clubhouse drawing was less effective at collecting member design input and engaging members than anticipated. Some members took advantage of the opportunity to engage in a quiet, individual activity, some applied and further discussed ideas from the evening, and others prepared to leave. Three rather than four exercises may have been more appropriate for the session. Alternatively, a collage activity based on images from other Clubhouses may be more engaging for members and useful for data collection.

#### 4.3.5. Research Team Reflections

The research team debrief after the participatory session documented several lessons and suggested future improvements to the exercises. Observations noted about training, exercise development, and administration by students aiming to inform future research assistants are reported here:

***Train to be inclusive, welcoming, and supportive***

- *Learn about and practice communicating clearly and respectfully with people of varying cognitive and physical abilities*, especially when providing instructions, answering questions, and offering assistance with exercises (e.g., offering to write comments on sticky notes when members could not).
- *Inquire about and practice using appropriate language* (e.g., members, not patients).
- *Identify opportunities to interact with the population.* Student research assistants said they would have felt more confident during the session with prior experience.
- *Arrange for participant transportation, food, and any other needs during the session.*

***Exercise Development***

- *Refer to and adapt existing participatory design research exercises and best practices.*
- *Develop work-oriented exercises that can easily be completed in a fixed time period.*
- *Create and test more exercises than needed.*
- *Include members and staff in exercise development, testing, and administration (and ideally data entry and analysis)*. Familiar faces were also helpful to attendees who initially preferred not to communicate with strangers.
- *Allow for some redundancy in data collection across exercises to improve reliability*. Member control over engagement means allowing missing responses. Vary task types and required levels of interaction with peers, staff, and the research team.
- *Begin with a short, simple, and engaging exercise*. Conduct longer or (minimally) complex exercises that generate important data early but not first in the session.

***Exercise Administration Preparation***

- *Test exercises from instructions through data entry and analysis*, then revise and repeat. Confirm that responses align with data collection goals.
- *Create detailed supply lists, timelines, and procedures* for set-up, exercise administration, and clean-up so team focus remains on the exercises and supporting participants during the session. Visit the space in advance and address any anticipated challenges.
- *Practice, practice, practice.* Rehearse all exercises, revise exercises and procedures, and repeat. Assign tasks to each research team member, with “back-up” as needed.

***Exercise Administration***

- *Wear friendly name tags and appropriate clothing*.
- *Stick to the schedule, but be flexible*. Prioritize member needs and experiences.
- *Ask participants about their work* to subtly confirm exercise instructions were clear.
- *Briefly check in with research team members* during and in-between exercises.
- *Emphasize Member expertise and the value of their input to inform the design process.*

## 5. Discussion

This case study examined use of participatory design research exercises to (1) document member needs, preferences, and priorities regarding future Clubhouse design and (2) meaningfully engage adults with SMI early in the Clubhouse design process in a manner aligned with Clubhouse principles. Member responses identified information about former Clubhouse services and activities needed by the Group, as well as member needs, preferences, and priorities to inform future Clubhouse design. Results focused on aesthetics and ambience; safety and security; ease of use and maintenance; adaptability, flexibility, and accessibility; transportation; preferred spaces and furnishings; and prioritizing space needs. Many of the resulting topics overlapped with existing MBH literature, especially the desire for de-institutionalized and homelike environments, affording choice and control, facilitating access to nature, and providing a smoking area [41,42,43,44,52,53,56]. Transportation comments also aligned with built environment and health literature that offers considerations for site selection and design. Clubhouse location likely affects member and staff travel time and reliance on vehicles; access to public transportation, affordable housing, employment, healthy food, and educational, social, and recreational opportunities; walkability and opportunities for physical activity; and safety [62,63].

Few studies gather information from building users with mental illness to inform the design of their facilities. Based on study results, participatory design exercises offer a useful and meaningful mechanism for engaging members in the Clubhouse design process, extending inclusive Clubhouse Model principles to facility design. Exercises were designed and administered in alignment with Clubhouse Model principles. Members were engaged as collaborative partners, rather than “research subjects,” in exploring their knowledge of and experiences with Clubhouses and mental illness following participatory literature [4,60]. Exercises afforded choice, control, and ownership and accommodated a range of abilities, comfort levels with verbal communication, and willingness to share information with the research team. Informal observations and feedback from members and the Group suggested that the type of information collected was useful to inform design, and that members felt included and engaged. The approach centered member strengths, enabled members to control their engagement level, and provided an opportunity for members to meaningfully contribute to the Clubhouse design process.

### 5.1. Limitations

The present study was not without limitations. First, although the participatory approach enabled collection of a substantial amount of qualitative open-ended data from 16 people in 90 min, the small, voluntary, and one-time sample was not representative of members or adults with SMI. While the Group pointed out that many attendees had been and were likely going to be frequent Clubhouse visitors, a representative sample is needed. A larger sample also enables identification of any potential trends in responses by member characteristics such as age, sex, cultural background, or diagnoses [41,45]. Multiple participatory sessions, rather than a one-time session, are also necessary. Second, the participatory design exercises used relied largely on Group and design professional input. Adapting existing reliable and validated tools to the Clubhouse population and incorporating more design features from the MBH literature may generate more reliable and valid responses to inform design. Third, members completed the exercises but did not participate in analysis or interpretation of results. Evaluation of the complete participatory design process requires member engagement in all three phases including results interpretation and design implementation. Future research is necessary to formally test the feasibility and effectiveness of using the complete participatory design process with Clubhouse members. Fourth, peer influences on responses and engagement during the session may have occurred, and members and staff may have only shared positive comments with the research team [3,4]. Follow-up interviews with members are needed to enable a collaborative member-checking process and allow individual participants to reflect on their responses [4]. Systematic evaluation of participatory exercise engagement and effectiveness is needed beyond informal observations and participant feedback.

### 5.2. Future Directions

The Clubhouse literature [1] lacks studies focused on the spaces and places in which the Clubhouse Model is implemented. Future work examining effects of and interactions between Clubhouse design and location features, informed by the MBH literature, on member outcomes is needed. Post-occupancy evaluations [64] and longitudinal studies of representative Clubhouse facilities can identify supportive design features specific to Clubhouse environments. Comprehensive, reliable, and valid design evaluation tools specific to Clubhouses must also be developed and tested to document cumulative effects of Clubhouse design in context. Existing tools from the MBH literature [65] can be adapted for use in Clubhouse settings, and informed by principles of universal design, trauma-sensitive design, and restorative design [52]. Restorative design elements [55,66], including biophilic design that emulates aspects of nature in the built environment [67], offer additional guidance and research questions for Clubhouse members, staff, researchers, and designers.

Further development and testing of participatory design research exercises will also increase the comprehensiveness of responses and usefulness to project stakeholders and design professionals. Evaluating the complete participatory design process with researchers, members, staff, and design professionals to inform best practices can facilitate more widespread and meaningful inclusion of members in the design process. Future work on Clubhouse environments and participatory design processes can also inform development of evidence-based design guidelines specific to the Clubhouse Model that can guide organizations working to open new or renovate Clubhouse facilities. Lastly, persons with different SMI diagnoses may respond differently to Clubhouse design features and participatory design processes. Experiences can also vary by age, race, sexual orientation, gender identity, and intersections of these member identities [68,69]. Inclusive and representative research aligned with Clubhouse Model principles is needed to identify design features beneficial to all Clubhouse members.

## 6. Conclusions

Participatory design exercises offer a promising approach to documenting design needs, preferences, and priorities and meaningfully engage adult members with serious mental illness in the Clubhouse design process. The approach aligns with inclusive Clubhouse Model principles. Findings from member input can inform not only project stakeholders, but future Clubhouse design and research. Collaborating and co-designing facilities with members via all participatory design phases may reveal additional strategies to leverage the Clubhouse built environment to further improve the success of the Clubhouse Model and member outcomes.

## Figures and Tables

**Table 1:** Average priority rankings of future Clubhouse spaces completed by members.

Must = 1 (1–1.5)		Should = 2 (1.6–2.5)		Could = 3 (2.6–3)	
Space	Average	Space	Average	Space	Average
Meeting room	1.0	Classroom	1.6	Balcony	2.6
Smoking area	“	Dining room	“	Bike storage	“
Reception desk/lobby	1.2	Lockers	“	Gallery	“
Kitchen	“	Stairs	“	Audio-visual	2.8
Copy center	“	Café/snack bar	1.8	Member housing	3.0
Restrooms	“	Covered outdoor space	“	Visitor lodging	“
Parking	“	Custodial storage	“		
Work room	1.4	Delivery area	“		
Computer room	“	Porch	“		
Admin. Office	“	Donation storage	2.0		
Director’s office	“	Exercise room	“		
Elevator	“	Garage/storage shed	“		
Laundry/mudroom	“	Garden	“		
		Library/reading room	“		
		Mechanical room	“		
		Recreation area	“		
		Retail space	“		
		Drop-off area	2.2		
		Arts and crafts studio	2.4		
		Banking office	“		
		Employment office	“		
		Fitting room	“		
		Lounge	“		
		(Any) outdoor space	“		

**Table 2:** Potential benefits of participatory exercises for the Members, Group, and Students.

Observed Benefits According to:	Selected Comments
*Clubhouse Members* Contributed to design process All engaged at some level Felt included	“*Educational and entertaining*” “*Atmosphere of excitement*” “*When can we do this again?*” “*When will this be built?*”
*Clubhouse Working Group*	
Informative and identified priorities Inclusion of Members in design Treated Members with dignity	“*We wanted to make sure a new building supported our needs, we just didn’t realize how much that support can depend on our space and location.*”
*Architecture Students*	
Information available Importance of user input Motivation to design well	“*I would completely change my design*” “*What… architects don’t always talk to users?!*” “*We did so little but it mattered so much*”

## Data Availability

De-identified data presented in this study are available on request from the corresponding author.

## Supplementary Materials

The following are available online at https://www.mdpi.com/article/10.3390/ijerph19116743/s1, Form S1: Clubhouse Questionnaire, Form S2: Clubhouse Space Priorities, Table S1: Questionnaire Results, Table S2: Design Project Feedback Results.
