# Supplementary material for: Engaging U.S. Adults with Serious Mental Illness in Participatory Design Research Exercises
Source: Int J Environ Res Public Health. 2022 May 31;19(11):6743. doi: 10.3390/ijerph19116743 (PMC9180822; doi:10.3390/ijerph19116743)
Supplement: Supplementary file 1 [file ijerph-19-06743-s001.zip › ijerph-1639887-supplementary.pdf]

Supplementary Material for

Engaging U.S. Adults with Serious Mental Illness in Participatory Design Research Exercises

Kimberly A. Rollings

[1] School of Architecture and the Department of Psychology, University of Notre Dame, Notre Dame, IN 46556, USA; kirollin@umich.edu
[2] Health and Design Research Fellow, Institute for Healthcare Policy and Innovation, University of Michigan, Ann Arbor, MI 48109, USA

1. Clubhouse Questionnaire (Form S1)
2. Clubhouse Space Priorities (Form S2)
3. Questionnaire Results (Tables S1a-S1d)
4. Design Project Feedback Results (Table S2)

1. Clubhouse Questionnaire (Form S1)

The Working Group, professors, and students [redacted] are documenting information about the design of Clubhouse buildings and spaces. Please complete the following questions. All responses will remain confidential.

1a. I am a (circle one): Former Clubhouse Member Future Clubhouse Member
Staff member Other: ____________

1b. How much time, on average, did you spend at the former Clubhouse each week?

________ days per week, and ________ hours per day on average

1c. During what hours did you typically visit the Clubhouse? Circle all that apply.

a. Morning b. Afternoon c. All day

2. How did you travel to and from the Clubhouse? Circle all that apply.

a. Car (own/borrowed)
b. Carpool
c. Bus
d. Walk
e. Bike
f. Train
g. Taxi
h. Other: ____________

3a. What work units and services did the Clubhouse offer? Circle all that apply.

a. Clerical/office
b. Attendance records
c. Accounting/financial
d. Food prep/serving
e. Maintenance/cleaning
f. Thrift store/retail
g. Gardening
h. Artistic activities
i. Recreational/social events
j. Education
k. Employment
l. Housing
m. Outreach
n. Transportation
o. Health services
p. Case management
q. Other: ____________

3b. List the work unit(s) in which you were involved: ____________

3c. Which additional units or services should a future Clubhouse offer? List all that apply:

____

4a. Did any work units or activities share a space in the Clubhouse? If yes, which ones?

____

4b. If yes, was sharing a space a problem? Why or why not? ____________

5a. What was your *favorite* space in the Clubhouse? Circle one.

a. Entryway/lobby
b. Stair hall
c. Office
d. Work room
e. Dining room
f. Kitchen
g. Snack bar
h. Library
i. Classroom
j. Outdoor space
k. Elevator
l. Restroom
m. Parking lot
n. Other: ____________

5b. Why is that space your *favorite* space? ____________

Go to page 2 (back)

5c. How much time, on average, did you spend each day in that space? ________________________

6a. List your *least* favorite space in the Clubhouse: ____

6b. Why is the space your *least* favorite? ____

____

6c. How much time did you spend each day in that space? _________________________________

7. Think about the Clubhouse *building* and *spaces*. What makes the Clubhouse facility different from a healthcare facility? *(Think about the lighting, sounds, odors, colors, entry, materials, furnishings, the way you are greeted, how you feel when you are there, etc.)* ____

____

____

____

8a. What was your favorite activity in the Clubhouse? Why? _________________________________

____

8b. What was your *least* favorite activity in the Clubhouse? Why? _____________________________

____

9a. What were your responsibilities and roles in the former Clubhouse? __________________________

____

9b. What made your responsibilities easier (*e.g., enough supplies, space, light, help*)? ____________________

____

9c. What made your responsibilities more difficult (*e.g., <u>not</u> enough supplies, space, light, help*)?

____

10. What would make travel to and participation in Clubhouse activities easier for you *(financial resources, transportation, weather, cost, job hours, other reasons…)?* **Why?** __________________________________

____

____

Personal information (optional; all individual responses remain anonymous and confidential):

- **I.** **Age** ______________ years
- **II.** **Gender** ______________
- **III.** **Education** (*circle one*): some high school   high school   some college   college
- **IV.** **If you been diagnosed with a mental illness, will you please share the diagnosis with us?**

  ____

***** Thank you for your participation! *****

2. CLUBHOUSE SPACE PRIORITIES (Form S2)

Buildings are expensive. In order to understand what spaces and activities are critical to a new building, including a Clubhouse, clients often need to prioritize and cut items from their "wish lists." Please look at the following list of potential Clubhouse spaces. With your group, decide whether each space listed here is a "must have" (1), "should have" (2), or "could have" (3) space. You may use the cards and sort them into pockets if that is helpful.

1. MUST: A required space, without which a new Clubhouse could not function.
2. SHOULD: Should really be in a new Clubhouse, but could be eliminated if necessary.
3. COULD: Would be very nice to have, but is more of a luxury.

____ Reception desk & lobby	____ Exercise Room	____ Porch
____ Work Room	____ Gallery	____ Bike Storage
____ Dining Room	____ Recreation Area	____ Garden
____ Café/Snack Bar	____ Lounge	____ Balcony
____ Kitchen	____ Library	____ Outdoor Space/Yard
____ Banking Office	____ Computer Room	____ Garage/Storage Shed
____ Director's Office	____ Retail Space	____ Delivery Area
____ Employment Office	____ Fitting Room	____ Drop-off Area
____ Meeting Room	____ Donation Storage	____ Parking
____ Restrooms	____ Visitor Lodging	____ Laundry/Mud Room
____ Administrative Office	____ Locker Room	____ Custodial Storage
____ Copy Center	____ Member Housing	____ Covered Outdoor Space
____ Classroom	____ Stairs	____ *Other* ________________
____ Audiovisual Studio	____ Elevator	____ *Other* ________________
____ Arts and Crafts Studio	____ Mechanical Room	____ *Other* _______________

You may add as many additional room types as needed, just be sure to also make a "card" for the space as well. Use the back of this sheet to explain your decisions, if there's time.

3. QUESTIONNAIRE RESULTS

Table S1a. Previous Clubhouse services and Member responsibilities according to Member questionnaire responses.

**Clubhouse services**		**Member responsibilities**
2 No response	2 Outreach	6 No response
9 Food prep/serving	1 Artistic activities	7 Computer work: Menu (2), attendance records (3), newsletter (1), computer classes (1)
6 Clerical/office	1 Case management	3 Variety; *"I had a choice of activity," "Help out"* [where needed]
6 Education	1 Garden	2 Fundraising, reach out, banking, statistics, teaching, leadership
5 Accounting/financial	1 Health services	2 Retail: Cashier (2) and snack bar inventory (1)
5 Maintenance/cleaning	1 Housing	1 Education lab
3 Recreational/social events	1 Transportation	1 Cooking, serving lunch, and more
3 Thrift store/snack bar (retail)	1 Fellowship/counseling	1 *"I took on a role of trust both with the staff and the members"*
2 Employment	1 Library	

Numbers indicate the number of Member responses that included that item.

Table S1b. Factors that made Member responsibilities easier or more difficult, according to questionnaire responses.

**What made responsibilities easier?**	**What made responsibilities difficult?**
9 No response	8 No response + 1 not legible response
3 Help/more help (1-from *"Students wanting to get their GEDs"*)	2 Space/rooms too small
2 More space, enough seating for lunch	2 Not enough supplies
1 *"Ease of maintenance was important"*	1 Could always use help
1 Discussion	1 [Computer] log-in problems
1 Food delivery	1 Me/depression
1 Working/available computer	1 *"People not showing up on time for their shift"*
1 *"Working with someone else"*	1 *"People just hanging out in the education lab"*
1 *"Knowing that I was making a difference"*	1 *"[People] not having any mental health education"*

Numbers indicate the number of Member responses that included that item.

Table S1c. Members' favorite and least favorite Clubhouse activities and why, according to questionnaire responses.

**Favorite Clubhouse Activities**	**Why**	**Least Favorite Clubhouse Activities**	**Why**
7 No response	--	7 No response	--
1 Visit/tour	- *"Visiting, taking the tour, I didn't want to be in Clubhouse"*	3 Mopping & Cleaning	- *"Cleaning; I don't especially like to clean;" "Cleaning bathrooms, but I did it willing as it was important to be clean"*
1 Cooking	- *"Because I could quietly serve others"*		
1 Meal setup & dishwashing	--	1 Housekeeping	--
1 Newsletter & clerical duties	- *"I got to know more about individuals and their stories."*	1 Computer	--
		1 Policing cigarette butts	--
1 Fundraising	- *"Selling candy bars, car washes, chicken sales"*		
1 Helping others	- *"It kept me out of my own head."*	1 Cook out	--
1 Get togethers	--	1 Not much to do	--
1 Trips	--	1 Talking in front of large groups	- *"Really nothing to do w/ the space-except for the acoustics were bad in many rooms"*
1 Bingo	--		
1 Tutoring	--		

Numbers indicate the number of Member responses that included that item.

3. QUESTIONNAIRE RESULTS (continued)

Table S1d. Favorite and least favorite Clubhouse spaces and reasons, according to Member questionnaire responses.

Favorite Clubhouse Space	Why	Least Favorite Space	Why
5 No response		7 No response	
3 Outdoor space	Smoking	2 Restroom	*- "Cleaning the bathrooms"*
3 Entryway/ Lobby	*- "Because this is how to show [visitors] how we feel about Clubhouse",* greet guests, phone messages	1 Cleaning	*- "Takes too long and is sort of hard work"*
3 Dining room	*- "There was a lot of conversation, eating, and caring there. And game playing"*	1 Office	--
3 Snack bar	*- "I liked working the cash register"*	1 Kitchen	*- "Dull"*
3 Classroom	--	1 Thrift shop	*- "Although when I did participate it was fulfilling"*
2 Computer room	*- "Where I did the most fun jobs"; "place where I could learn, teach, and create such things as the newsletter, cards, track attendance, and other activities involving club organization"*	1 Chairs	*- "There were no easy chairs I think"*
2 Kitchen	*- "Small so not as overwhelmed b/c smaller group"*	1 Smoking porch	*- "I am an anti-smoker"*
1 Garden	*-Relaxing*	1 Storage garage	*- "Dark, stinky, lonely"*
1 Library	--	1 Meeting space	*- "Big meetings & big meeting space tee hee= what was worse were big meetings in small meeting space"*
1 Elevator	--		
1 All	*- "The whole building. I would do anything [any Clubhouse task in any space] to get out every day"*		

Numbers indicate the number of Member responses that included that item.

4. DESIGN PROJECT FEEDBACK RESULTS

Table S2. A summary of Member feedback on student Clubhouse design proposals.

**Future Clubhouse spaces, furnishings, and equipment**	- Lobby/reception area (visible from entry)
- Café/snack bar with an ice cream bar, storage, and a juke box	- Kitchen and pantry
- Recreational space/game room (include clean-up rules)	- Classroom (math and other activities)
Pool table (3), ping pong, foosball, Wii, variety of games	- Thrift store
- Social room: Rocking chairs, gliders, bean bag chairs	- Smoking area (3)
- Small dining room in addition to large (& offer a variety of foods)	- Garden (veggies & shrubs)
- Copy space for printing news, letters, brochures	- Windows with a view
- Paper shredder in the office	- Public transportation and parking
- Indoor sunroom/garden room/atrium/courtyard	- Van (carpool)
- Indoor water feature; pond (2) for fish and meditation	- Reading room/library
- Fitness room (2): bikes, treadmill, indoor pool and hot/whirlpool	- Television lounge
- Laundry area (& clean thrift store clothing)	- Bank
- Two work areas & computers for all work units (esp. kitchen group)	- Space for creative work
- Outdoor area for car washes and other fundraisers	- Piano or organ
**Ease of use and maintenance**	**Architecture/appearance**
- Easy cleaning/maintaining of interior & exterior spaces	- Combination of two common local "old" styles
- One story easier to maintain than two	- Felt homey
- Snow removal considerations	- Previous Clubhouse: Liked the woodwork
- Custodial storage on each floor	- Openness
- Lots of water hose outlets	- Lots of light like the former dining room
- Adequate electrical outlets	- Avoid having any space feel utilitarian
- Pantry/food storage near service (delivery) entrance	
- Garden within close proximity of the kitchen	
**Safety & Security**	**Adaptability, flexibility & accessibility**
- Lockable front door	- Ability to play music throughout via a sound system
- Supervision in the fitness area	- Moveable partitions, e.g., for expanded dining/to create smaller spaces
- Be forced to walk through different spaces (surveillance & sociability)	- Universally designed accessible entrances
- First aid area	
- Lockers for secure storage of personal belongings	

Numbers indicate the number of Member responses that included that item. No number indicates one Member response.
